# Cost-minimization analysis in a blind randomized trial on small-incision versus laparoscopic cholecystectomy from a societal perspective: sick leave outweighs efforts in hospital savings

**DOI:** 10.1186/1745-6215-10-80

**Published:** 2009-09-04

**Authors:** Frederik Keus, Trudy de Jonge, Hein G Gooszen, Erik Buskens, Cornelis JHM van Laarhoven

**Affiliations:** 1 Department of Surgery, Diakonessenhuis, Bosboomstraat 1, 3582 KE Utrecht, The Netherlands; 2 St. Elisabeth hospital, Hilvarenbeekseweg 60, 5022 GC Tilburg, The Netherlands; 3 Department of Surgery, University Medical Center, Heidelberglaan 100, 3584 CX Utrecht, The Netherlands; 4 Julius Center for health sciences and primary care, University Medical Center, Heidelberglaan 100, 3584 CX Utrecht, The Netherlands; 5 Department of Surgery, University Medical Center St. Radboud, Geert Grooteplein-Zuid 10, 6525 GA Nijmegen, The Netherlands; 6 Current address: Department of Surgery, University Medical Center St Radboud, Geert Grooteplein-Zuid 10, 6525 GA Nijmegen, The Netherlands; 7 Current address: Department of Epidemiology, University Medical Center, Hanzeplein 1, 9713 GZ Groningen, The Netherlands

## Abstract

**Background:**

After its introduction, laparoscopic cholecystectomy rapidly expanded around the world and was accepted the procedure of choice by consensus. However, analysis of evidence shows no difference regarding primary outcome measures between laparoscopic and small-incision cholecystectomy. In absence of clear clinical benefit it may be interesting to focus on the resource use associated with the available techniques, a secondary outcome measure. This study focuses on a difference in costs between laparoscopic and small-incision cholecystectomy from a societal perspective with emphasis on internal validity and generalisability

**Methods:**

A blinded randomized single-centre trial was conducted in a general teaching hospital in The Netherlands. Patients with reasonable to good health diagnosed with symptomatic cholecystolithiasis scheduled for cholecystectomy were included. Patients were randomized between laparoscopic and small-incision cholecystectomy. Total costs were analyzed from a societal perspective.

**Results:**

Operative costs were higher in the laparoscopic group using reusable laparoscopic instruments (difference 203 euro; 95% confidence interval 147 to 259 euro). There were no significant differences in the other direct cost categories (outpatient clinic and admittance related costs), indirect costs, and total costs. More than 60% of costs in employed patients were caused by sick leave.

**Conclusion:**

Based on differences in costs, small-incision cholecystectomy seems to be the preferred operative technique over the laparoscopic technique both from a hospital and societal cost perspective. Sick leave associated with convalescence after cholecystectomy in employed patients results in considerable costs to society.

**Trial registration:**

ISRCTN Register, number ISRCTN67485658.

## Background

Langenbuch's classical cholecystectomy has been the gold standard for over a century [1]. Since the mid 1970's surgeons began shortening their incisions because of a presumed quicker convalescence [2,3]. Soon thereafter, laparoscopic cholecystectomy (LC) was introduced, and rapidly expanded around the world [4]. The popularity of this procedure was partly based on an appealing technological innovation as well as industry driven motives and not primarily a result of an evidence-based approach [5].

Analysis of evidence in Cochrane reviews shows no difference regarding primary outcome measures (mortality and complications) between the three operative techniques of cholecystectomy (open, small-incision and laparoscopic) [6-8]. In absence of clear clinical benefit based on these meta-analyses it may be interesting to focus on the resource use associated with the available techniques. We performed a single blind randomized clinical trial focusing on a secondary outcome: costs. In a previous paper we emphasized intrinsic validity of this trial, proved reproducibility of results from other trials and showed generalisability in a general teaching hospital.[9]

The costs of LC and small-incision cholecystectomy (SIC) have been compared in six randomized trials. [10-15] These available studies are inconsistent in outcome and conclusions, use different perspectives and most of the trials suffer methodological shortcomings.

The research question is whether there is a difference in costs from a societal perspective between small-incision and laparoscopic cholecystectomy using a blind randomized approach. In a detailed cost analysis attention has to be paid to both direct and indirect costs as well as the perspective of the analysis. Furthermore, cost prices, budget prices and tariffs have to be distinguished.

## Methods

In meta-analyses we found no major differences in clinical outcome measures (mortality, complications, conversions, hospital stay, and convalescence) between LC and SIC for patients with symptomatic cholecystolithiasis.[8] We also found no differences considering pulmonary function, health status, and cosmesis. [16,17] Costs are a secondary outcome measure and ultimately may be a decisional factor. This paper focuses on cost-minimization analysis.

Medical Ethics Committee approval for this single-centre trial was obtained in September 2000. Between January 2001 and January 2004, all patients referred to our surgical outpatient's clinic with symptomatic cholecystolithiasis (confirmed by ultrasonography) were considered for inclusion in this study.

### Inclusion- and exclusion criteria

Inclusion criteria were male or female patients with symptomatic cholecystolithiasis, aged 18 years or older at recruitment, reasonable to good health (ASA I or II), no known relevant allergies, and a signed informed consent letter.

Exclusion criteria were age younger than 18 years, choledocholithiasis (icterus, acholic faeces and/or bilirubine of twice normal range), cholangitis, known pregnancy, moderate to severe systemic disease (ASA III and higher), known cirrhosis of the liver, history of abdominal malignancy, previous upper abdominal surgery (precluding a laparoscopic approach), psychiatric disease, or a reasons (e.g. lack of knowledge of the Dutch language) that might make follow-up or completion of questionnaires unreliable.

Obesity was not an exclusion criterion. Recovery after successful endoscopic treatment of choledocholithiasis was not a contra-indication. Acute cholecystitis was excluded.

### Randomization

A random number table was used for generation of the allocation sequence [18] and the allocation concealment was guaranteed by using sealed envelopes. Patients were randomized after induction of anesthesia. An employee of the secretary office opened an envelope. Details were recorded in a case record form. Otherwise the procedure was recorded as 'trial cholecystectomy'.

### Surgical procedures

All patients had a standard anesthesia regime. Premedication, medications for induction and continuation of anesthesia, as well as respiration during surgery were standardized. Residents (from 2^nd ^year on) performed most of the operations. In case of technical difficulties either trial technique could be converted to open cholecystectomy (OC). Wounds were covered with standard wound dressings.[19] In this way blinding of patients, nurses, and ward physicians was achieved. Postoperative analgesics and medication for nausea were standardized.

Open introduction was performed in all laparoscopic cholecystectomies. Pneumoperitoneum was created with an intra-abdominal pressure up to 12 mmHg. Three trocars for instruments were inserted. The dissection of the cystic artery and cystic duct, identifying Calot's triangle, was performed using a three points 'flag' technique [20] The cystic duct and artery were clipped and transected. All instruments were reusable.

In concordance with literature a cut-off point of 8 cm was used to differentiate between SIC and OC.[14,19,21-26] The incision was placed over the musculus rectus abdominis. No special equipment was used. Access to the peritoneum was obtained by a 'muscle splitting' technique. The gallbladder was dissected by a 'fundus-first' technique. The cystic duct and artery were ligated and the gallbladder was removed. Posterior and anterior fascias were closed separately. If the length of the incision exceeded 8 cm, the operation was considered to be a conversion to OC.

### Postoperative protocol

Early oral intake and mobilization were encouraged. Patients left the hospital as soon as they were able to do so. Incidental 'social' reasons for lengthening of hospital stay (by a few days) were accepted. Hospital stay was defined as the number of postoperative nights in hospital. For logistic reasons, we were not able to blind the surgeon during follow-up. Follow-up was standardized after 2 weeks, 6 weeks and 3 months. Patients were encouraged to resume work as soon as possible.

### Analysis and sample size

In meta-analyses no differences in patient-relevant outcomes appear to be present between these two techniques. Assuming no differences in primary outcome measures, sample size calculation was based on anticipated differences of costs. The direct costs of the first 50 patients in the trial were calculated so as to estimate the likely range of differences in costs and their standard deviations. On this basis, we estimated that 120 patients per group would be needed to detect a difference of 10% in direct costs using an α of 0.05 and a β of 0.9.

Analyzing differences in costs due to complications between both techniques would require another sample size including thousands of patients in order to possibly find significant results. Consequently, differences in complication costs were therefore not statistically tested in our study. However, these costs were reported to illustrate their impact on total costs.

### Statistics

All data were stored in a case record form (Access^®^) based on a patient-linked trial registration number. A double data entry was performed. Comparisons were made on an intention to treat basis. Calculations were made using SPSS 11.0^®^.

The chi-square test was used for dichotomous outcome. Normality of data was checked using the Kolmogorov-Smirnov test. [27] Levene's test was used for checking equality of variances. When the condition of normality and equal variances was met, the t-test for independent data was used; otherwise the nonparametric Mann-Whitney U test for independent data was used.

### Methods of cost analysis

As cost items that are equally present in both groups do not contribute to differences, it can be argued that these can be left out of consideration. On the other hand all costs contribute to the total amount and the incremental value. Therefore, we strived for reporting costs in detail. [28] For each cost item, hospital costs, overhead costs and consultants' costs were included if appropriate (Table 1).

**Table 1:** List of cost items used in calculations of total costs. For each cost item hospital costs, overhead costs and consultants costs are included if appropriate.

	**Item**
**Preoperative**	General practioner

	First visit to outpatient clinic (20 min)

	X-thorax *

	ECG

	Blood examinations

	Consultation pulmonologist (36 min)

	Consultation cardiologist (22.65 min)

	Consultation internist (30 min)

	ERCP (30 min)

	Ultrasound * (10 min)

**Operative**	Hospital operating room per minute

	Anaesthesiologist per minute^#^

	Surgeon per minute

	Surgical resident per minute

	Laparoscopic instruments - reusable

	Laparoscopic instruments - disposable

**Admittance**	Ultrasound localization of gallbladder

	Blood gas analysis (Åstrup)

	Spirometry analysis

	One night hospital stay

	One night medium care (including intensivist)

	Intensive care (with mechanical breathing)

	Intensive care (without mechanical breathing)

	Pathology examination

**Follow-up**	Outpatients visit (10 min)

**Complications**	Ultrasound drainage (10 min)

	Blood culture

	Blood transfusion

	Urologist outpatients visit (30 min)

	Gastroscopy (30 min)

	MRCP *

	CT abdomen *

	CT thorax *

	CT angiography *

	CT cerebrum *

	MR cerebrum *

	Re-laparotomy

	Emergency department visit

	Ultrasound duplex

* Time spend by radiologists at diagnostic procedures was estimated at 10 minutes

^# ^In our hospital anaesthesiologists are responsible for two operations at a time. Therefore, 140 euro (per hour) was calculated for two operations resulting in 1.17 euro per minute per operation.

Time spend by consultants (for diagnostic procedures) in brackets.

All costs were calculated in euros (2004). All direct medical costs were summarized in different categories including costs due to complications (admittance, operative, outpatients' clinic, and complications).

As there are no relevant or significant differences in clinical outcome [8,16] or quality-of-life [17], a cost-minimization analysis seems most appropriate. Afterwards, differences in costs can be balanced with other (thus far unknown) differences in outcome.

In evaluating costs, it is important to be complete in accounting for all items.[29] Therefore, in general a societal perspective is recommended. [30-32] In a societal perspective all costs are included in the analysis (patient, hospital and losses in production), irrespective of the stakeholder incurring the costs or who benefits from treatment. Moreover, today's limited health care budgets warrant proper economic evaluation of treatments, especially when incidences and impact on economy are high (like in symptomatic cholecystolithiasis).

### Definitions of cost categories

#### Direct medical costs

costs resulting from outpatient clinic, hospital admittance, surgery, complications, etcetera.

#### Direct non-medical costs

costs arising from outside health care immediately related to treatment (e.g. traveling costs from patients). The problem is that it is very difficult to estimate these costs accurately in all patients. We assume that these costs are equal for both groups. Moreover, since the vast majority of patients have an uncomplicated recovery, these costs are assumed not to contribute importantly to total costs.

#### Indirect non-medical costs

costs due to loss of productivity related to employment status of patients. These costs arise from loss of productivity caused by sick leave, disabled for work or mortality. We decided to use the friction cost method. [31-36]

### Tariffs, cost prices, budget prices and overhead costs

In cost assessments tariffs, cost prices and budget prices have to be distinguished. Tariffs are costs that are calculated for insurance companies. Tariffs are nearly always different from real costs. Budget prices on the other hand are figures used for internal (hospital) calculations, and do not reflect real costs either. Cost prices are real costs for procedures and are usually not used in hospital administrations. These cost prices can be calculated but are sometimes difficult to retrieve.

Apart from these differences there are costs for depreciation and interest. Different methods are used and no uniform guidelines exist how to estimate these overhead costs, neither do arguments exist that one method is superior over the other. In our hospital a percentage incremental value is added to every cost price. We applied this method in our study.

### Description of procedures and measurement of resources

In this cost-minimization analysis, all resources were prospectively recorded. A visit to the general practitioner, diagnostic examinations, and costs due to preoperative outpatient clinic visits were counted. Hospital stay was counted as the number of overnights stay. Medication use is included in admittance cost prices. Operative costs and the costs of anesthesiologists were calculated according to operating room occupancy. Standard materials and equipment used in the operating room including costs associated with cleaning and sterilisation are included in hospital costs for surgery. The costs of the surgeon and resident were calculated from the time of incision to last suture. In laparoscopic instruments extra laparoscopy-specific materials like clips and endobags were calculated, but monitor, gass-insufflator, and camera were not calculated as these were considered present. In the small-incision procedure no extra equipment other than standard instruments is needed. In follow-up the time of the surgeon was calculated. Finally, if complications occurred, all extra costs were included. Costs of consultants were calculated using the national agreed honorarium (140 euro per hour) (Table 1).

### Sensitivity analyses

Several sensitivity analyses were performed to yield an impression of the effect of changes on total costs. Variables were considered if the costs were appreciable and a change in the costs of the variable could be possible and clinically relevant:

1. The influence of the use of disposable instead of reusable laparoscopic instruments on total costs.

2. Influence of reduction of time back-to-work by one week on total costs.

3. Influence of the reduction of hospital stay by one night on total costs.

## Results

**Figure 1:**
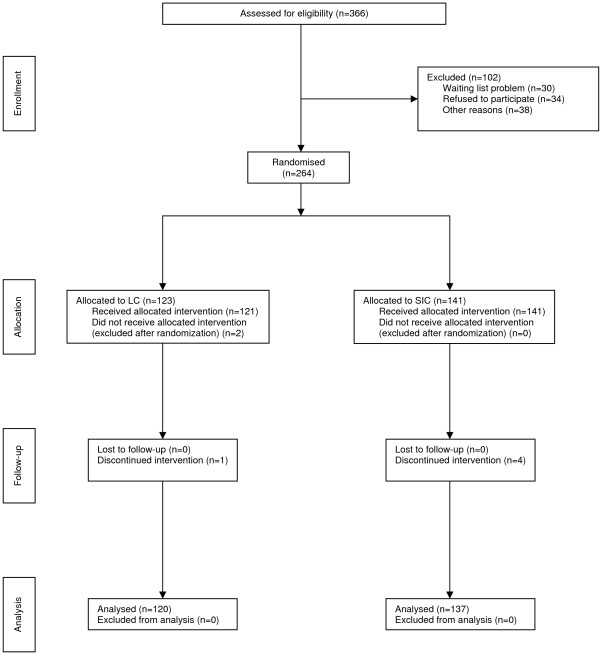
**Revised consort statement diagram showing the flow of participants through each stage of the randomized trial **[37].

All trial patients were included and operated between January 2001 and March 2004. Leaving unwilling and excluded patients out of consideration, 366 patients visiting the outpatient clinic of the hospital for symptomatic cholecystolithiasis fulfilled the inclusion criteria and were initially included in the trial. A total of 102 patients were not randomized for a variety of reasons (Figure 1). [37] After randomizing 264 patients, another 7 patients were excluded for the following reasons: unwilling to continue in the trial (n = 2), intra-operative suspicion of malignancy (n = 2), transfer to a non-surgical ward (n = 1), inadvertent participation in multiple trials (n = 1), and inadequate Dutch language skills (n = 1). A total of 257 patients were left for analysis (LC:120 and SIC:137).

The two treatment groups did not differ regarding age, sex, body mass index (BMI), and American Society of Anesthesiologists (ASA) classification (table 2). None of the 257 patients were lost to follow-up and resources of all patients could be determined.

**Table 2:** Patient characteristics.

	**Laparoscopic cholecystectomy** **(n = 120)**	**Small-incision cholecystectomy** **(n = 137)**	**Statistical analysis**
Male/Female	31/89	30/107	p = 0.459

Age			
mean (SD)	48.4 (14.1)	48.5 (14.0)	p = 0.974
median (range)	49 (17-77)	48 (18-80)	

BMI			
mean (SD)	27.5 (4.8)	27.9 (4.6)	p = 0.500
median (range)	26.8 (18.5-45.9)	27.2 (18.0-43.3)	

ASA			
I	82 (68.3%)	91 (66.4%)	p = 0.855
II	38 (31.7%)	46 (33.6%)	

The numbers of converted procedures, hospital stay, the number of residents performing the procedure, and the number of intra-operative and postoperative complications were not significantly different. Operative time was significantly shorter in the small-incision group (difference 11 minutes, 95% confidence interval (CI) 6 to 17 minutes; p < 0.001) (table 3). The total costs for both treatment groups including all 257 patients are summarized (Table 4).

**Table 3:** Comparison of clinical results.

	**Laparoscopic cholecystectomy** **(n = 120)**	**Small-incision cholecystectomy** **(n = 137)**	**Statistical analysis**
Mortality	0	0	

Complications	21	16	p = 0.119
intra-operative	5	3	p = 0.363
postoperative	16	13	p = 0.331

Failed symptom relief	11 (9.2%)	14 (10.2%)	p = 0.777

Operative time (min):			
mean (SD)	71.9 (25.8)	60.4 (18.3)	p < 0.001
median (range)	68.5 (26-215)	60.0 (29-105)	

Conversion rate	14 (11.7%)	22 (16.1%)	p = 0.312

Operative team:			

surgeon-resident	15 (12.5%)	21 (15.3%)	p = 0.515
resident-surgeon	84 (70.0%)	100 (73.0%)	p = 0.596
resident-resident	21 (17.5%)	18 (13.1%)	p = 0.331

Hospital stay*			
mean (SD)	2.4 (4.6)	3.1 (12.4)	p = 0.560
median (range)	1 (1 - 36)	2 (1 - 144)	

Hospital stay* (without 1 extreme value) mean (SD)	2.1 (3.4)	2.0 (2.4)	p = 0.877

Number of patients with:			
1 night stay postoperative	67 (55.8%)	62 (45.3%)	p = 0.091
2 nights stay postoperative	38 (31.7%	56 (40.9%)	p = 0.127

* hospital stay: in postoperative nights

**Table 4:** Overview of costs with all patients included and without one extreme value in each group (in euro).

	**Laparoscopic cholecystectomy**	**Small-incision cholecystectomy**	**Difference in costs** **(per patient)**	**significance**
**All patients included (intention-to-treat)**	**(n = 120)**	**(n = 137)**		

Direct costs	305760	452654		
*(per patient)*				
				
- Outpatients clinic related	*2548*	*3304*	-756	P = 0,006*
*(per patient)*				
- Operation theatre related				
*(per patient)*				
- Admittance related	54293	62627		
*(per patient)*	*452*	*457*	-5	P = 0.640
- Complications related				
				
Indirect costs	133406	123404		
*(per patient)*	*1112*	*901*	211	P < 0.001*
				
Total costs	67972	81707		
*(per patient)*				
	*566*	*596*	-30	P = 0.342
	50089	184917		
	(n = 50)	(n = 51)		
	172005	155024		
	*3440*	*3040*	400	P = 0.315
	477765	607678		
	*3981*	*4436*	-454	P = 0.737

**Without one outlier in each group**	**(n = 119)**	**(n = 136)**		

Direct	272584	282683		
*(per patient)*	*2291*	*2079*	212	P = 0.006*
				
- Outpatient clinic related	53980	62273		
*(per patient)*	*454*	*458*	-4	P = 0.669
- Operation theatre related	130796	121856	203	P < 0.001*
*(per patient)*	*1099*	*896*		
- Admittance related	67421	81131		
*(per patient)*	*567*	*597*	-30	P = 0.346
- Complications related	20388	17423		
				
Indirect	(n = 50)	(n = 51)		
	172005	155024		
*(per patient)*	*3440*	*3040*	400	P = 0.315
				
Total costs	444589	437706		
*(per patient)*	*3736*	*3218*	518	P = 0.034*

* significant difference

Negative differences in costs favour the laparoscopic procedure and positive differences in costs favour the small-incision technique.

There is an important difference in direct costs. The difference is caused by differences in costs due to complications and rendering total costs in favor of the laparoscopic procedure.

The difference in operation theatre cost is in favor of the SIC group however. Operation theatre costs are over 23% more expensive in the LC group compared to the SIC group (LC: 1112 euro compared to SIC: 901 euro; difference 211 euro, p < 0.001). Indirect costs are higher in the laparoscopic group.

Data of costs are non-Gaussian distributed, importantly influenced by one extreme outlier. Therefore, although intention-to-treat is violated, the results excluding one outlier in each group are also shown (Table 4). Results in the cost categories admittance and outpatients' clinic do not change. There still is a difference in operation theatre costs in favor of the SIC group (difference 203 euro; 95% CI 147 to 259 euros). In the total costs, there is a difference in favor of the SIC group. All patients were operated using reusable laparoscopic instruments and costs were calculated accordingly.

In order to be able to compare uncomplicated LC and SIC procedures, costs were also calculated excluding all complicated cases. There still are differences present in the operation theatre related costs (difference 199 euro; 95% CI 139 to 259 euro; p < 0.001) and in total direct costs (difference 139 euro; 95% CI 42 to 237 euro; p = 0.006). No significant differences were observed in other direct cost categories, indirect costs, and total costs.

In order to estimate the influence of indirect costs on total costs we performed calculations only including employed patients (Table 5). Indirect costs appear to amount over 60% of total costs in both groups.

**Table 5:** Overview of costs with employed patients only (in euro).

	**Laparoscopic cholecystectomy** **(n = 120)**	**Small-incision cholecystectomy** **(n = 137)**	**Difference in costs** **(per patient)**	**significance**
60 and older	26	26		
Employed (male/female)	50	51		
Unemployed/unknown	44	60		

**Employed**	n = 50	n = 51		
Direct	(37.7%)	(38.4%)		
	104003	96454		
*(per patient)*	*2080*	*1891*	189	P = 0.055
				
Indirect	(62.3%)	(61.6%)		
	172005	155024		
*(per patient)*	*3440*	*3040*	400	P = 0.315
- Average period before return to work (in weeks (SD)):	4.2 (2.3)	3.7 (2.0)		P = 0.298
				
Total	276008	251477		
*(per patient)*	*5520*	*4931*	589	P = 0.179

Total indirect costs: 327029 euro (n = 101)

Average period before return to work: 4.0 (SD: 2.2) weeks

Average indirect costs per patient per week: 820 euro

Negative differences in costs favour the laparoscopic procedure and positive differences in costs favour the small-incision technique.

We performed a sensitivity analysis assuming the use of disposable instead of reusable laparoscopic instruments. Calculations show increase of operation costs resulting in differences of approximately 960 euro in favor of the SIC group (95% CI 912 to 1024 euro; p < 0.001).

A sensitivity analysis assuming a decrease in work leave by 1 week for the employed patients (n = 101) in this trial results in savings of 82790 euro. In another sensitivity analysis assuming a decrease in hospital stay by 1 night for the employed patients (n = 101) in this trial results in savings of 22980 euro.

## Discussion

When no differences in primary outcomes are found, consequently, several secondary outcome measures like operative time, hospital stay, and time to recovery as well as costs can be chosen as focus for a trial. Most of these secondary outcome measures are incorporated in a total cost assessment. Our cost analyses show that SIC is more expensive compared to LC when all patients are included (intention to treat, Table 4). Excluding one outlier from analyses in each group, total costs per patient are higher in the LC group (p = 0.034). This difference is caused by a difference in operative costs (difference 203 euro; 95% CI 147 to 259 euro; p < 0.001) and a difference in indirect costs (p = 0.315). When all complicated cases are excluded, direct costs (p = 0.006) and operative costs (199 euro, p < 0.001) per patient remain higher in the LC group (using reusable laparoscopic instruments).

The problem in reporting costs are non-Gaussian distributions. Following intention-to-treat principles, complicated cases should be included to obtain an objective impression of absolute costs. Using means results in a biased impression of falsely increased measures as a consequence of skewed data, while using medians would ignore complicated cases since in cholecystectomy about 80% of operations are uneventful procedures (Table 4). In our trial one extreme outlier occurred. Differences in techniques have to be distinguished from random variations. Meta-analyses demonstrate no differences in complications between laparoscopic and small-incision cholecystectomy. We therefore believe that differences in complication costs should be considered random variations. Moreover, our trial was not powered to detect differences in complication costs and these results should be considered a spurious finding and should therefore not be statistically tested at all. Reporting costs excluding one outlier in each group might therefore be more correct as it incorporates complication costs but prohibits distortion of total costs by random extreme outliers.

There are several problems in analyzing and pooling cost results from different studies. First of all, costs are reported in different ways including different cost items. A second problem is that different points of views are taken. These different perspectives make comparison of studies difficult. A third problem is the difference in validity of the cost assessments, defined by the details in which costs are calculated. More detailed analyses are known for more reliable estimates, while less detailed studies cause severe bias.[28,29] A fourth problem in comparing studies is that there may be considerable differences in local costs. Specific items in cost analyses differ from one country or even setting to another. A fifth and probably most important problem are cultural differences. There are wide variations in convalescence (and return to work) between different cultures depending on a multitude of causes, like social security and cultural habits.[38,39] As multiple factors cause heterogeneity, pooling results seems inappropriate and one may only draw general conclusions from individual studies.

In literature six trials report costs and lack of methodological quality was present in several trials.[10,12,14,40] In some trials methodology of cost assessment was very limited described.[12,40] Outpatients' costs [11,12] and indirect costs.[10-12,14] are excluded in several studies making overall (societal) comparison of techniques incomplete. Retrospective analyses [14] or expert settings [11,14] raise questions on reliability and generalisability. In our trial surgical residents performed 86% of the operations.

The trials by Calvert and Nilsson are high quality trials.[11,13] Unfortunately, outpatients' clinic costs and indirect costs are not included in the trial by Calvert.[11] Additionally, median hospital stay and estimated operative time (instead of individual data) are used for calculations. They concluded that hospital costs using the laparoscopic technique were 29% higher.[11] The trial by Nilsson is a high quality multi-centre trial. However, standardization of procedures is less biased and more uniform in a single-centre trial compared to a multi-centre trial. Costs are reported in medians (ignoring outliers and complications). This study found lower direct costs and higher indirect costs for the SIC group.[13]

Some conclusions of differences in costs were based on differences in hospital stay [10] or convalescence.[13,14] However, in meta-analysis no differences were found in hospital stay and convalescence between both techniques.[8] The differences in indirect costs in our trial should be considered as random variations caused by random differences in age and sex between both groups in the employed patients: calculated friction costs per hour per employee are higher for male and for higher aged employees.

Remarkably, the trials with lack in methodological or cost assessment quality [10,14,40] favor the laparoscopic technique, while the trials with high methodological quality or more detailed cost assessments [11,13] favor the small-incision technique. This linkage between unclear/inadequate methodological quality to significant overestimation of beneficial effects and underreporting of adverse effects is in concordance with other studies.[8,41,42]

Different parties have different interests in cost analyses. Though, all perspectives belong to our society. Advantages of a certain therapy in a societal perspective should be given more importance compared to other perspectives not including all cost categories. It provides the most comprehensive assessment and is most relevant for national policy decisions. Implementation at a local level, however, may require to also taking into account a hospital perspective as financial consequences will become visible.

Feasibility of ambulatory cholecystectomy [43,44] and the wide range in return to work from a few days to 12 weeks raises questions on potential savings. Possibilities for future savings by reduction of hospital stay (direct costs), irrespective of the operating technique for cholecystectomy, were compared to savings by reduction of sick leave (indirect costs). Assuming 21000 cholecystectomies in the Netherlands, reducing hospital stay by one night (50%) in every patient would result in potential savings of 4.8 million euro in the Netherlands annually. Assuming that 50% of the cholecystectomy patients are employed (Table 5), reducing sick leave by one week (25%) in every employed patient would lead to savings of 8.6 million euro on a national basis annually. Based on these hypothetical figures it seems easier to achieve savings by earlier return to work instead of reducing hospital stay. Moreover, as more than 60% of costs of employed patients are caused by sick leave it is more logical to focus on this cost category.

Assuming 21000 cholecystectomies in the Netherlands and an employment ratio of 50%, calculations of sensitivity analyses on hypothetical savings were performed considering change in policy from disposable to reusable laparoscopic instruments, change from LC to SIC, or reducing sick leave by one week. As a result savings of approximately 16 million, 4.2 million and 8.6 million euro respectively are possible on a national basis annually. However, conclusions have to be careful since calculations are hypothetical.

## Conclusion

In this single-centre trial with representative results and emphasis on methodological quality LC appears more costly: the procedural costs surpass those of SIC (and use of disposable instruments would only further increase this difference). Thus SIC is the preferred operative technique over LC both from a hospital and societal cost perspective.

Sick leave associated with convalescence after surgery results in considerable costs to society especially in the employed patient.

## Competing interests

The authors declare that they have no competing interests.

## Authors' contributions

FK and CJHML had full access to all of the data in the study and take responsibility for the integrity of the data and the accuracy of the data analysis. FK, CJHML, EB and HGG participated in study concept and design. FK, TJ and CJHML participated in the acquisition of data. FK, HGG, CJHML participated in the analysis and interpretation of data. KF and TJ drafted the manuscript. HGG, EB and CJHML were responsible for critical revision of the manuscript for important intellectual content. FK, EB and CJHML participated in statistical analysis. FK and CJHML obtained funding. FK, TJ and CJHML were responsible for administrative, technical, and material support. HGG and CJHML supervised the study.
